# Characterization of Human CD8 T Cell Responses in Dengue Virus-Infected Patients from India

**DOI:** 10.1128/JVI.01424-16

**Published:** 2016-11-28

**Authors:** Anmol Chandele, Jaturong Sewatanon, Sivaram Gunisetty, Mohit Singla, Nattawat Onlamoon, Rama S. Akondy, Haydn Thomas Kissick, Kaustuv Nayak, Elluri Seetharami Reddy, Haroon Kalam, Dhiraj Kumar, Anil Verma, HareKrushna Panda, Siyu Wang, Nasikarn Angkasekwinai, Kovit Pattanapanyasat, Kulkanya Chokephaibulkit, Guruprasad R. Medigeshi, Rakesh Lodha, Sushil Kabra, Rafi Ahmed, Kaja Murali-Krishna

**Affiliations:** a ICGEB-Emory Vaccine Center, International Center for Genetic Engineering and Biotechnology, Aruna Asaf Ali Marg, New Delhi, India; b Pediatric Pulmonary Division, Department of Pediatrics, All India Institute of Medical Sciences, Ansari Nagar, New Delhi, India; c Department of Research and Development, Faculty of Medicine Siriraj Hospital, Mahidol University, Bangkok, Thailand; d Emory Vaccine Center, Emory University School of Medicine, Atlanta, Georgia, USA; e Department of Pediatrics, Division of Infectious Disease, and Emory Vaccine Center, Emory University School of Medicine, Atlanta, Georgia, USA; f Immunology Group, International Center for Genetic Engineering and Biotechnology, Aruna Asaf Ali Marg, New Delhi, India; g Translational Health Science and Technology Institute, Faridabad, Haryana, India; h Department of Microbiology, Faculty of Medicine, Siriraj Hospital, Mahidol University, Bangkok, Thailand; i Department of Microbiology and Immunology, Emory University School of Medicine, Atlanta, Georgia, USA; j Department of Pediatrics, Faculty of Medicine, Siriraj Hospital, Mahidol University, Bangkok, Thailand; k Department of Urology, Emory University School of Medicine, Atlanta, Georgia, USA; Washington University School of Medicine

## Abstract

Epidemiological studies suggest that India has the largest number of dengue virus infection cases worldwide. However, there is minimal information about the immunological responses in these patients. CD8 T cells are important in dengue, because they have been implicated in both protection and immunopathology. Here, we provide a detailed analysis of HLA-DR^+^ CD38^+^ and HLA-DR^−^ CD38^+^ effector CD8 T cell subsets in dengue patients from India and Thailand. Both CD8 T cell subsets expanded and expressed markers indicative of antigen-driven proliferation, tissue homing, and cytotoxic effector functions, with the HLA-DR^+^ CD38^+^ subset being the most striking in these effector qualities. The breadth of the dengue-specific CD8 T cell response was diverse, with NS3-specific cells being the most dominant. Interestingly, only a small fraction of these activated effector CD8 T cells produced gamma interferon (IFN-γ) when stimulated with dengue virus peptide pools. Transcriptomics revealed downregulation of key molecules involved in T cell receptor (TCR) signaling. Consistent with this, the majority of these CD8 T cells remained IFN-γ unresponsive even after TCR-dependent polyclonal stimulation (anti-CD3 plus anti-CD28) but produced IFN-γ by TCR-independent polyclonal stimulation (phorbol 12-myristate 13-acetate [PMA] plus ionomycin). Thus, the vast majority of these proliferating, highly differentiated effector CD8 T cells probably acquire TCR refractoriness at the time the patient is experiencing febrile illness that leads to IFN-γ unresponsiveness. Our studies open novel avenues for understanding the mechanisms that fine-tune the balance between CD8 T cell-mediated protective versus pathological effects in dengue.

**IMPORTANCE** Dengue is becoming a global public health concern. Although CD8 T cells have been implicated both in protection and in the cytokine-mediated immunopathology of dengue, how the balance is maintained between these opposing functions remains unknown. We comprehensively characterized CD8 T cell subsets in dengue patients from India and Thailand and show that these cells expand massively and express phenotypes indicative of overwhelming antigenic stimulus and tissue homing/cytotoxic-effector functions but that a vast majority of them fail to produce IFN-γ *in vitro*. Interestingly, the cells were fully capable of producing the cytokine when stimulated in a T cell receptor (TCR)-independent manner but failed to do so in TCR-dependent stimulation. These results, together with transcriptomics, revealed that the vast majority of these CD8 T cells from dengue patients become cytokine unresponsive due to TCR signaling insufficiencies. These observations open novel avenues for understanding the mechanisms that fine-tune the balance between CD8-mediated protective versus pathological effects.

## INTRODUCTION

Dengue disease is becoming a global epidemic, with nearly 40% of the world's population at risk for transmission of one or more of the four dengue virus (DENV) serotypes, and the mosquito vectors that transmit these viruses continue to spread to other parts of the world. Approximately 390 million human dengue infections are estimated to occur annually, with 100 million clinical disease cases, the symptoms of which range from fever to hemorrhage and shock, often leading to death, especially among children. India is estimated to have the highest dengue burden in the world, but there is minimal to no information on the human cellular immune response to dengue virus infections from India. Currently there are no available antivirals. Several vaccines are under research, development, or clinical trials, and a live, recombinant, tetravalent dengue vaccine was recently licensed and approved for use in Mexico, Brazil, El Salvador, Paraguay, and Philippines. Thus, there is a compelling need for a better understanding of the immunology of the human host to dengue virus during clinical disease.

Both innate and adaptive responses are involved in dengue virus immunity, but CD8 T cells are of particular interest because of their role in eliminating virus-infected targets through cytotoxic effector function and thus are also of great interest from a vaccination perspective.

CD8 T cells are also important in dengue, because they have been implicated both in protection against dengue and in causing cytokine-mediated immune pathology (1–16). Because the dengue-specific memory T cells secrete cytokines upon *in vitro* stimulation with heterologous viral antigen (3, 13), it was suspected that the “cytokine storm” induced by activated T cells may contribute to the immunopathology of dengue. These suspicions were further strengthened by the observations that CD8 T cell expansion peaks before or around the time of the peak of clinical disease and that the frequencies of activated CD8 T cells and cytokine-producing cells were somewhat higher in patients with severe forms of the disease (5, 8). More recent studies, on the other hand, highlight an HLA-linked protective role for CD8 T cells in dengue (1, 7, 12, 14–18). Despite many of these elegant studies, significant gaps remain in our understanding of CD8 T cell properties during the febrile phase of dengue disease. Therefore, in this study, we addressed the following questions. What is the overall expansion of the different CD8 T cell subsets in dengue patients? What changes occur in the gene expression profiles of the activated CD8 T cells from dengue patients? What are the phenotypes of these different CD8 T cell subsets? What fraction of each of these activated CD8 T cell subsets produce gamma interferon (IFN-γ) in response to dengue virus antigens?

By using a combination of phenotypic, functional, and transcriptomic approaches, our studies revealed that both HLA-DR^+^ CD38^+^ and HLADR^−^ CD38^+^ CD8 T cell subsets expanded massively in dengue patients. Both CD8 T cell subsets expressed markers indicative of overwhelming antigenic stimulus and proliferation, tissue homing, and cytotoxic-effector functions, with the HLA-DR^+^ CD38^+^ subset being more robust in these effector qualities. The expression profiles of these activated CD8 T cells were strikingly similar to those of whole blood or peripheral blood mononuclear cells (PBMCs) analyzed from dengue patients from different geographical regions across the continents. Surprisingly, despite this strong effector phenotype, we found that only a minute proportion of these massively expanding activated effector CD8 T cells were capable of producing IFN-γ cytokine when stimulated *in vitro*. Transcriptomics studies revealed that these cells downregulated pathways involved in T cell receptor (TCR) signaling, and functional studies confirmed that these TCR signaling insufficiencies might contribute to their inability to produce IFN-γ. These results open novel directions for a better understanding of the mechanism by which CD8 T cell-mediated protective versus pathological effects are fine-tuned during the time of febrile illness and open novel therapeutic avenues for dampening inflammation, especially in infections like dengue.

## MATERIALS AND METHODS

### Patient cohort.

Patients diagnosed with dengue disease either at All India Institute of Medical Sciences (AIIMS), New Delhi, India, or at Siriraj Hospital, Bangkok, Thailand, were enrolled in this study between the years 2011 and 2015. Dengue virus infection was confirmed by a combination of methods, including serotype-specific reverse transcription (RT)-PCR, as well as several other diagnostic tests (NS1 enzyme-linked immunosorbent assay [ELISA] and dengue virus-specific IgG and IgM [ELISA or dipstick] tests). The WHO 1997 classification was used to characterize the patients as having dengue fever (DF), dengue hemorrhagic fever (DHF), or dengue shock syndrome (DSS) (19). Healthy young adults (18 to 25 years old) were used as controls. All the studies were preapproved by institutional review boards, and informed consent was obtained from the enrolled patients. Information about the patient cohort is provided in Table 1.

**TABLE 1:** Summary of dengue patients analyzed in this study^*a*^

Parameter	Value	
	AIIMS, New Delhi, India	Siriraj, Bangkok, Thailand
Total no. of patients	108	45
No. of males/females	56/52	34/11
Age (yr) [range (avg)]	1.2–14 (8.9)	6–18 (12)
No. of days post-onset of clinical symptoms [range (avg)]	2–10 (4.6)	3–11 (6.2)
No. of patients DENV NS1 positive	92	23
No. of patients DENV IgM positive	16	22
No. of patients DENV PCR positive	82	36
No. of patients serotyped	80	17
DENV-1	3	1
DENV-2	76	4
DENV-3	1	6
DENV-4	0	6
No. of patients with classified disease grade	108	45
DF	44	30
DHF	21	15
DSS	43	0
Serological status and disease grade of dengue patients (no.)		
Primary dengue	48 (25 DF; 10 DHF; 13 DSS)	15 (13 DF; 2 DHF)
Secondary dengue	60 (19 DF; 11 DHF; 30 DSS)	18 (11 DF; 7 DHF)
Not assigned	0	3 (0 DF; 3 DHF)
Not done	0	9 (6 DF; 3 DHF)

a One hundred eight dengue patients from New Delhi and 45 patients from Bangkok were analyzed in the study.

### IgM/IgG ratio evaluation.

Patients who do not show any detectable levels of serum dengue-specific IgM and IgG (seronegative) and those who show levels of dengue-specific IgM greater than those of IgG by a ratio ≥1.2 are generally considered to have primary dengue infections. Patients who show IgG only or IgM levels lower than those of IgG by a ratio of ≤1.2 are usually considered to have secondary dengue infection (19). In order to characterize primary and secondary dengue cases in our study based on these criteria, we performed capture ELISA for both IgM and IgG using Panbio dengue virus IgM and IgG capture ELISA kits (Alere; product codes 01PE10 and 01PE20).

### PBMC and plasma isolation.

PBMCs and plasma were isolated as described previously (20). Briefly, blood samples were collected in Vacutainer CPT tubes (Becton Dickinson [BD]). Plasma samples were isolated from the CPT tubes and preserved at −80°C. The PBMCs were collected, washed extensively, and suspended in phosphate-buffered saline (PBS) containing 2% fetal calf serum (FCS) for immediate use or frozen in liquid nitrogen in FCS with 10% dimethyl sulfoxide (DMSO) for subsequent analysis.

### Analytical flow cytometry.

Cells were washed and stained for 30 min in ice-cold PBS containing 10% bovine serum albumin. All the antibodies were purchased from Becton Dickinson except CD71 (Biolegend; 334108), granzyme B (Invitrogen; MHGB04), Ki-67 (EBioscience; 11-5699-42), T-Bet (EBioscience; 50-5825-80), and ICOS (EBioscience; 46-9948-42). For intracellular protein staining, cells were permeabilized with Cytofix/Cytoperm buffer (Becton Dickinson), followed by staining for 60 min with antibodies that were diluted in Perm/Wash buffer (catalog no. 554723; Becton Dickinson). The fixable viability dye eFluor 780 (EBioscience; 65-0865-18) was used for excluding dead cells during analysis. Flow cytometry data acquisition was performed either on a FACSCanto II or an LSR-II (Becton Dickinson). Flow cytometry data were analyzed using FlowJo software (TreeStar Inc.). Phenotypes and functions were analyzed among gated CD8 T cells, defined as cells that expressed both CD3 and CD8. Absolute cell numbers per milliliter volume of blood were calculated using the BD Trucount Tubes bead system (BD; 340334) according to the manufacturer's protocol.

### *Ex vivo* stimulation of PBMCs.

PBMCs were cultured for 6 h with or without stimulation. The stimulations included a total of 511 15-mer peptides that overlapped by 10-mers that spanned the entire proteome of dengue virus serotype 2 (DENV-2) (kindly provided by BEI Resources). These peptides were reconstituted in DMSO and then combined into pools that represented each of the 10 dengue virus proteins (capsid, PrM, envelope, NS1, NS2A, NS2B, NS3, NS4A, NS4B, and NS5). Where indicated, more than one megapool was generated because of the large number of amino acids. The final concentrations of individual peptides at the time of stimulation were adjusted to 2 μg/ml. Cells were stimulated with peptides, along with costimulation using purified anti-human CD28 and CD49D (BD; 340957 and 340976). In situations where cells were polyclonally stimulated, pretitrated beads coated with anti-CD3 plus anti-CD28 antibodies (Dynabeads Human T-activator CD3/28 for T cell expansion and activation; Invitrogen; 11131D) or a mixture of phorbol 12-myristate 13-acetate (PMA) and ionomycin at a concentration of 1× (cell stimulation cocktail; EBioscience; 00-4970-03) was used. The cells were cultured for 2 h at 37°C, and then brefeldin A (GolgiPlug; BD; 555029) was added, followed by a further 4 h of culture. The cells were then harvested; surface stained with cocktail containing fixable viability dye (EBioscience; 65-0865-18), CD3 (Biolegend; 300424), CD8 (Biolegend; 301048), CD38 (BD; 562288), and HLA-DR (BD; 560896); and then fixed and permeabilized using a Cytofix/Cytoperm kit (BD; 554722). The cells were then stained with IFN-γ (BD; 554700) and CD69 (Biolegend; 310920) and analyzed on a BD Fortessa FACS Scan or BD Canto II.

### Fluorescence-activated cell sorter (FACS) sorting of naive and activated CD8 T cells.

PBMCs isolated from peripheral whole blood of dengue patients and healthy controls were stained with the relevant antibodies at 4°C for 30 min, washed thoroughly, suspended in PBS containing 2% FCS, and immediately sorted on a BD FACS Aria III (Becton and Dickenson) with high forward-scatter gates to account for the larger blasting effector lymphocytes. CD3^+^ CD8^+^ CD45RA^+^ CCR7^+^ naive CD8 T cells and CD3^+^ CD8^+^ HLA-DR^+^ CD38^+^ activated CD8 T cells were isolated to a purity of 99%.

### Gene expression analysis of CD45RA^+^ CCR7^+^ naive and HLA-DR^+^ CD38^+^ effector CD8 T cells.

The sorted cells were washed thoroughly, and the cell pellet was immediately suspended in TRIzol (Invitrogen) and stored at −80°C until RNA extraction. All the samples were processed simultaneously for RNA isolation by standard TRIzol RNA isolation procedures using glycogen as a carrier. Total RNA was evaluated by spectrophotometry to determine the quantity, protein concentration, and organic solvent contamination, and an Agilent 2100 bioanalyzer was used to determine RNA degradation. Two rounds of *in vitro* transcription were performed to amplify RNA, and then cRNA was labeled and hybridized on a human genome U133 plus 2.0 array (Affymetrix) by GSR Microarrays, Vanderbilt University. The chips were scanned on a seventh-generation GeneChip scanner 3000 (Affymetrix), and Affymetrix GCOS software was used to generate the raw intensity data file.

### Microarray data analysis.

The gene expression data in .CEL file format were converted and annotated using R software. Expression values lower than 20 were converted to a value of 20. For genes that were detected by multiple probes, only the one with the highest average expression value was kept for further analysis. A total of 19,738 genes were analyzed. For each gene, an average of the expression values in the naive CD8 T cells (CCR7^+^ CD45RA^+^) was used as the baseline for expression. To determine the statistical significance of the expression of each gene between the dengue and naive groups, a 2-tailed Student *t* test was used, and the *P* value was calculated with Microsoft Excel. Hierarchical clustering (HCL) and principal-component analysis (PCA) were performed using MultiExperiment Viewer (MeV) version 10.2. For HCL analysis, Pearson correlation for distance metric calculation and the average linkage method were used. For PCA, the median value was used as the centering mode. The Broad Institute's Molecular Signatures Database (MSigDB) version 5.1 (http://software.broadinstitute.org/gsea/msigdb) was used to determine the biological pathways in which the up- and downregulated genes were involved.

### GSEA.

Gene set enrichment analysis (GSEA) was performed using GSEA v2.2.2 (Java version 1.0), downloaded as a desktop tool from the Broad Institute (21). HLA-DR^+^ CD38^+^ CD8 T cells were compared to three data sets: GSE51808, which compares whole blood from patients with acute dengue and healthy subjects from Thailand (M. Kwissa et al. [22]), GSE43777, which compares PBMCs from patients with acute dengue and convalescent dengue patients from Venezuela (P. Sun et al. [23]), and GSE18090, which compares PBMCs from patients with acute dengue and nondengue patients from Brazil (E. J. Nascimento et al. [24]). Normalized enrichment score analysis was performed as previously described (25).

### Statistical analysis.

Unpaired two-tailed *t* tests with Welch's corrections were used to determine statistical significance.

### Accession number(s).

The original .CEL files were uploaded to the NCBI Gene Expression Omnibus (GEO) with accession number GSE84331.

## RESULTS

### Activation and expansion of CD8 T cell subsets during dengue infection.

**FIG 1:**
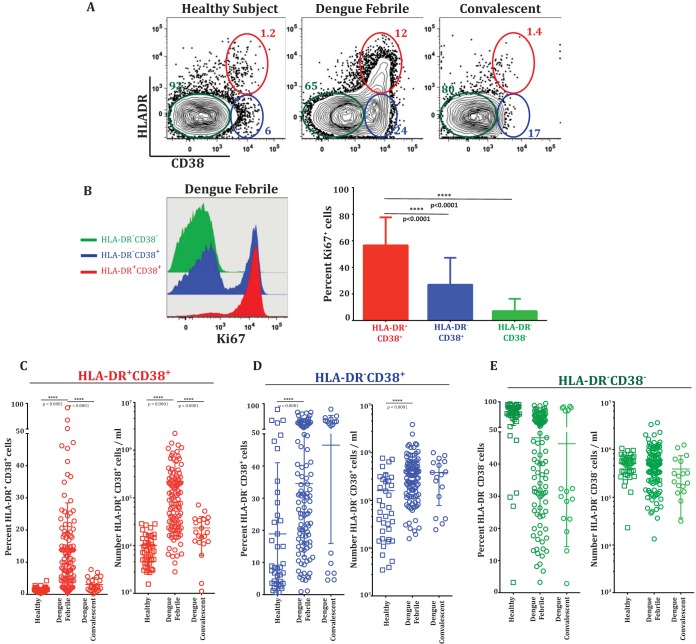
Activation and expansion of CD8 T cell subsets during dengue infection. (A) Representative flow cytometry plots of gated CD8 T cells in a naive healthy subject, an acute dengue fever patient, and a convalescent subject. CD8 T cells were further gated for their expression of HLA-DR and CD38. Note that both HLA-DR^+^ CD38^+^ double-positive (red circles) and CD38^+^ single-positive (blue circles) CD8 T cells expand in dengue fever patients. (B) (Left) Proliferation of individual HLA-DR^+^ CD38^+^, CD38^+^, and HLA-DR^−^ CD38^−^ subsets from a dengue fever patient shown in an overlay histogram of Ki-67. (Right) Average frequencies of Ki-67-positive cells in individual CD8 T cell subsets from dengue patients (*n* = 28). The bars indicate standard deviations. (C to E) Frequencies (percentages) and numbers per milliliter of blood of HLA-DR^+^ CD38^+^ (C), HLA-DR^−^ CD38^+^ (D), and HLA-DR^−^ CD38^−^ (E) CD8 T cell subsets from a large cohort of dengue fever patients from New Delhi, India. Values for individual persons are indicated. Horizontal lines represent the means of all data points, and bars indicate standard deviations. ****, *P* = 0.0001.

HLA-DR and CD38 coexpression is considered a hallmark feature of effector CD8 T cells in human viral infections (26). To characterize the expansion of these cells during dengue disease, we analyzed CD8 T cells derived from PBMCs from 153 confirmed dengue fever illness cases, 108 of which were from New Delhi, India, and 45 of which were from Bangkok, Thailand. The patient cohort is described in Table 1. Figure 1A shows an example of flow cytometric analysis of CD8 T cell subsets described as HLA-DR^+^ CD38^+^, HLA-DR^−^ CD38^+^, and HLA-DR^−^ CD38^−^. Ki-67 is a well-accepted marker for identification of cells that are actively proliferating or have recently proliferated. Analysis of Ki-67 in these three subsets of CD8 T cells showed that in dengue patients, approximately 60% of the HLA-DR^+^ CD38^+^ double-positive CD8 T cell population, and in some patients as high as 80%, were positive for the proliferation marker Ki-67 (Fig. 1B). The HLA-DR^−^ CD38^+^ single-positive CD8 T cell population also expressed Ki-67, albeit at lower frequencies, and the HLA-DR^−^ CD38^−^ double-negative population expressed very low levels of Ki-67. This suggests that both the HLA-DR^+^ CD38^+^ double-positive and the HLA-DR^−^ CD38^+^ single-positive CD8 T cells are highly proliferative *in vivo*, but the proliferative capacity was more dramatic in the HLA-DR^+^ CD38^+^ double-positive subset.

**FIG 2:**
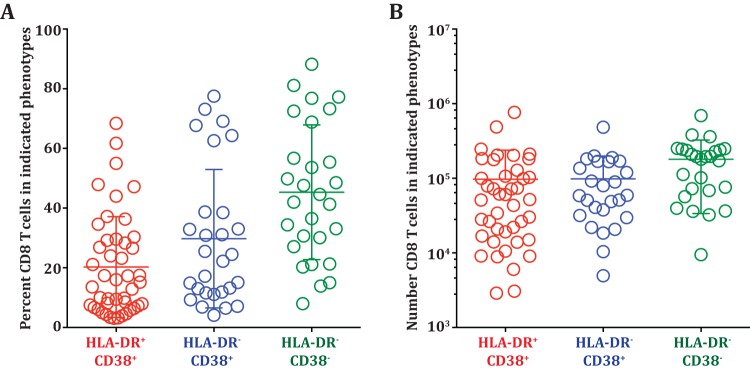
Expansion of CD8 T cell subsets in dengue patients from Thailand. (A) Frequencies of HLA-DR^+^ CD38^+^, HLA-DR^−^ CD38^+^, and HLA-DR^−^ CD38^−^ subsets in dengue patients from Bangkok, Thailand. (B) Absolute numbers of HLA-DR^+^ CD38^+^, HLA-DR^−^ CD38^+^, and HLA-DR^−^ CD38^−^ subsets in dengue patients from Bangkok, Thailand.

**FIG 3:**
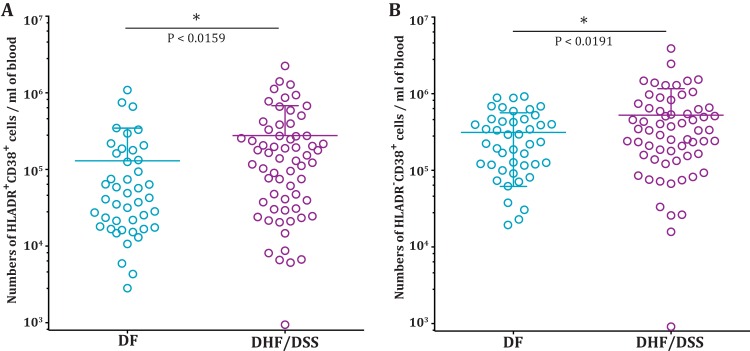
Expansion of HLA-DR^+^ CD38^+^ and CD38^+^ CD8 T cell subsets in DF and DHF/DSS. (A) Scatter plots showing numbers per milliliter of blood of HLA-DR^+^ CD38^+^ subsets in dengue fever and DHF/DSS. (B) Scatter plots showing numbers per milliliter of blood of HLA-DR^−^ CD38^+^ subsets in dengue fever and DHF/DSS. *, *P* = 0.05.

The frequency of the HLA-DR^+^ CD38^+^ CD8 T cells was generally low (average, 1.5%) in healthy subjects, but these cells expanded dramatically in the dengue patients (Fig. 1C, left). Their average frequency was about 15% of the total CD8 T cells, and in some patients, their frequencies reached as high as 80%. These HLA-DR^+^ CD38^+^ CD8 T cells average about 0.2 × 10^6^ cells per ml blood and reached as high as 1 × 10^6^ per ml blood in some of the dengue patients (Fig. 1C, right). The frequencies and numbers of these activated CD8 T cells declined in the convalescent phase. The frequencies (Fig. 1D, left) and numbers (Fig. 1D, right) of the HLA-DR^−^ CD38^+^ cells varied dramatically even among the healthy subjects (range, 1% to 80% of the CD8 T cells), although the fraction of healthy subjects with a high frequency of HLA-DR^−^ CD38^+^ CD8 T cells tended to be lower. Interestingly, our results showed that these HLA-DR^−^ CD38^+^ single-positive CD8 T cells also substantially increased in the dengue patients. The fact that the HLA-DR^−^ CD38^+^ cell subset also expanded in numbers, in addition to showing enrichment for Ki-67, suggests that the HLA-DR^−^ CD38^+^ cells are also likely to be antigen-specific proliferating effectors. The Ki-67-negative cells in each of the CD8 T cell subsets could have been activated and proliferated in the past and now have ceased proliferation (27). We next examined whether the expansion of the two CD8 T cell subsets was also seen in dengue patients from different geographical regions. Our analysis of dengue patients from Thailand showed that the average frequencies and numbers of the two activated CD8 T cell subsets (HLA-DR^+^ CD38^+^ and HLA-DR^−^ CD38^+^) were also similar in dengue patients from Thailand (Fig. 2A and B). Moreover, our results from India and Thailand are consistent with previous studies reporting expansion of HLA-DR^+^ CD38^+^ and HLA-DR^−^ CD38^+^ cells in dengue patients from Brazil (4) and Vietnam (6). The previous studies also suggested that the numbers of activated CD8 T cells were higher in dengue patients with severe disease. Although our study was not designed to extensively study disease severity, we also found that the numbers of both activated CD8 T cell subsets were moderately higher in patients with DHF/DSS than in patients with mild DF (Fig. 3A and B). Viral loads were not significantly different between the two groups, as shown in our previous study (28). From these data, we conclude that both HLA-DR^+^ CD38^+^ and HLA-DR^−^ CD38^+^ CD8 T cell subsets proliferate and expand massively in dengue patients. The expansion of these different CD8 T cell subsets is seen in dengue patients from different geographical regions of the world despite the expected population HLA differences.

### Phenotypic analysis of CD8 T cell subsets from dengue patients.

**FIG 4:**
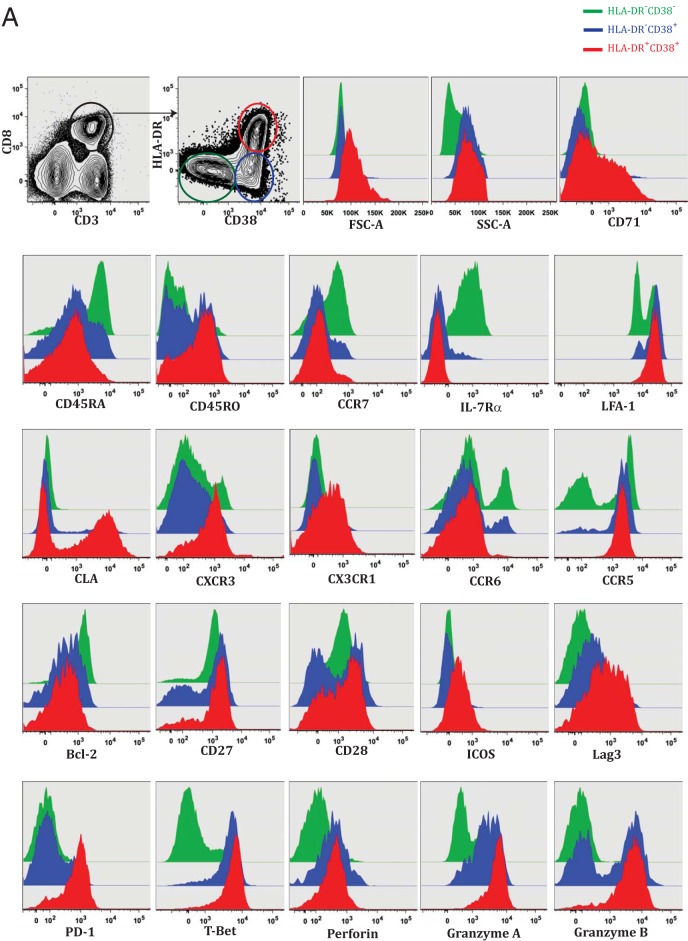

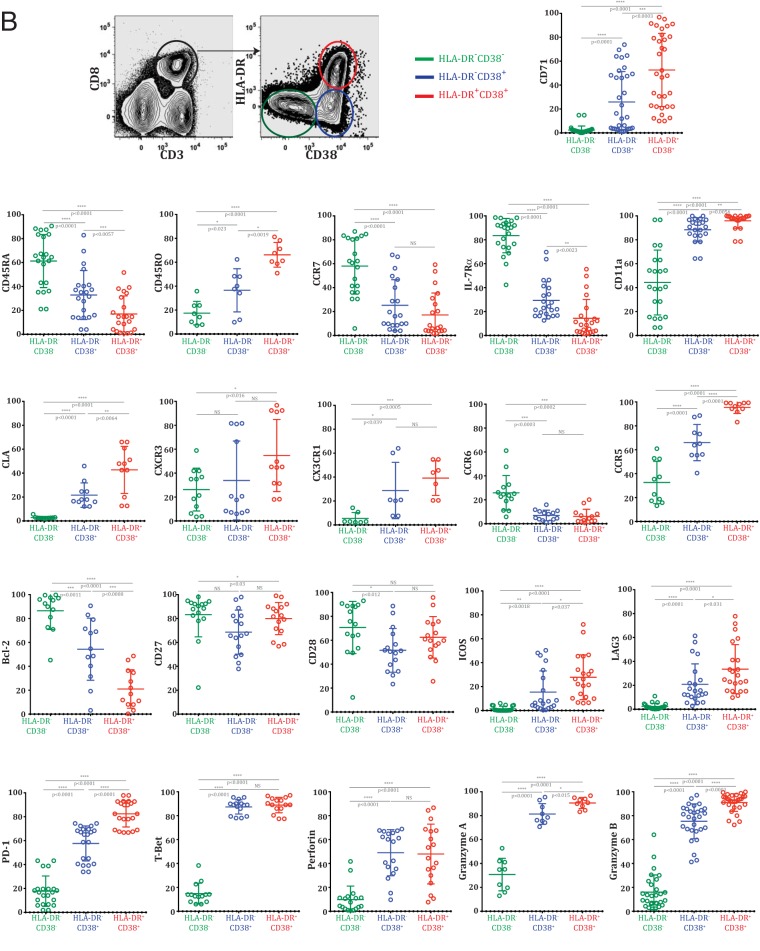
Phenotypic analysis of CD8 T cells from dengue patients. (A) Phenotypes *ex vivo* in HLA-DR^+^ CD38^+^, HLA-DR^−^ CD38^+^, and HLA-DR^−^ CD38^−^ subsets from dengue fever patients. The two plots at the top left show the gating strategy and the three subsets of CD8 T cells for the overlay histograms. (B) Data pooled from patients from Delhi and Bangkok to estimate the spread of each phenotype. *, *P* = 0.05; **, *P* = 0.01; ***, *P* = 0.001; ****, *P* = 0.0001; NS, not significant.

Despite the many elegant studies that have investigated CD8 T cell association with dengue disease or HLA-linked protection, a detailed understanding the phenotypes of these activated CD8 T cells in the febrile illness stage and how the phenotypes differ between CD8 T cell subsets is lacking. Hence, we performed a comprehensive immune phenotyping of the HLA-DR^+^ CD38^+^ double-positive, HLA-DR^−^ CD38^+^ single-positive, and HLA-DR^−^ CD38^−^ double-negative CD8 T cell subsets from the dengue patients. Figure 4A shows flow cytometric analysis of the phenotypes of these different CD8 T cell subsets, and the spread of the phenotypes in individual patients is shown in Fig. 4B. Both HLA-DR^+^ CD38^+^ double-positive and HLA-DR^−^ CD38^+^ single-positive CD8 T cell populations had high forward scatter, high side scatter, and upregulated CD71, which is a transferrin receptor involved in iron uptake among the proliferating cells. However, this activation was more pronounced in the HLA-DR^+^ CD38^+^ double-positive CD8 T cells. Both HLA-DR^+^ CD38^+^ and HLA-DR^−^ CD38^+^ CD8 T cells could egress the lymphoid organs (downregulated CCR7), were enriched with terminally differentiated effector cells (downregulated interleukin 7 receptor [IL-7R] alpha chain), increased cell-cell interactions (upregulated leukocyte function-associated antigen 1 [LFA-1]), and were programmed by a Th1/Tc1 differentiation pathway (upregulated T-bet). However, neither of the subsets represented early-stage effectors (downregulated Th17 lineage marker CCR6). We also found that the two subsets downregulated the anti-apoptotic molecule Bcl-2 and to some extent the costimulatory molecules CD28 and CD27.

The HLA-DR^+^ CD38^+^ CD8 T cell subset was more pronounced in the downregulation of CD45RA and the upregulation of CD45RO, suggesting that the subset was more activated. The subset was able to traffic to the skin and mucosa (upregulated cutaneous lymphocyte antigen [CLA]) and to adhere, survive, and be maintained in inflamed tissues (upregulated CX3CR1); represented the most terminally differentiated Th1 lineage effector CD8 T cells (upregulated CXCR3); and was favored to respond to antigenic stimulus or had recently been exposed to antigen *in vivo* (upregulated costimulatory molecule ICOS). Although the HLA-DR^−^ CD38^+^ subset had low expression of CLA, CXCR3, and CX3CR1, the subset upregulated RANTES/MIB1-b receptor (CCR5) expression, similar to the HLA-DR^+^ CD38^+^ subset. This suggests that both subsets have the ability to home to the tissues but the HLA-DR^+^ CD38^+^ subset has the full spectrum of homing receptors. The HLA-DR^+^ CD38^+^ CD8 T cells also expressed the markers, suggesting that they might have experienced massive antigenic stimulus *in vivo* (upregulated two strong negative costimulatory molecules, LAG-3 and PD-1). Moreover, almost all of these HLA-DR^+^ CD38^+^ CD8 T cells strongly upregulated perforin, granzyme A, and granzyme B, whereas only a fraction of the HLA-DR^−^ CD38^+^ subset expressed granzyme A and granzyme B, suggesting that both subsets are capable of cytotoxic effector functions but the HLA-DR^+^ CD38^+^ subset is much more efficient.

We conclude that both HLA-DR^+^ CD38^+^ and HLA-DR^−^ CD38^+^ activated CD8 T cells in dengue patients are massively expanding, express markers of tissue homing, and are equipped with cytotoxic effector functions. However, the HLA-DR^+^ CD38^+^ CD8 T cells demonstrate more pronounced full-spectrum effector phenotypes.

### IFN-γ production in CD8 T cell subsets during dengue virus infection.

**FIG 5:**
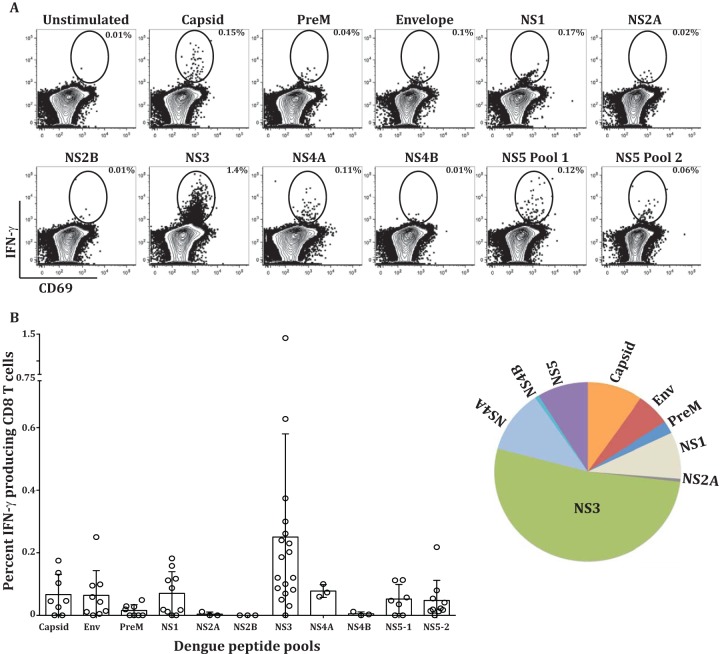

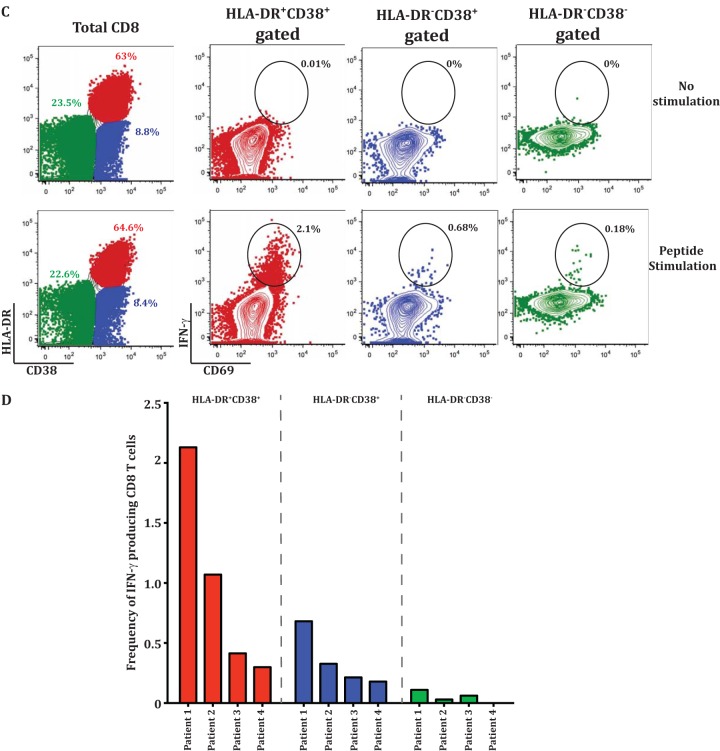
IFN-γ production by CD8 T cell subsets during dengue virus infection. PBMCs from dengue virus-infected patients were stimulated with overlapping peptide pools representing each of the 10 proteins of the dengue virus. Larger proteins were split into 2 or 3 pools, as depicted. (A) Flow cytometry plots showing percent IFN-γ-producing CD8 T cells by *ex vivo* stimulation. (B) Scatter plot showing frequencies of CD8 T cells producing IFN-γ in individual patients across the dengue proteome. The pie chart shows the immunodominance hierarchy of IFN-γ-producing cells by averaging IFN-γ production across each protein in 4 to 18 subjects from India and Thailand. (C) Frequencies of IFN-γ-producing cells in HLA-DR^+^ CD38^+^ (red), CD38^+^ (blue), and HLA-DR^−^ CD38^−^ (green) gated populations with and without the total NS3 peptide pool. (D) Frequencies of NS3 peptide pool-specific IFN-γ-producing cells in individual subjects in the indicated cell subsets.

Previous studies established that the CD8 T cells from dengue fever patients produce IFN-γ when stimulated with dengue virus peptides *in vitro*. Considering our results described above that show massive expansion, activation, tissue homing, and cytotoxic effector phenotypes of the two activated CD8 T cell subsets in dengue patients, it was of interest to examine what fraction of these cells produce IFN-γ, what is the breadth of the response, and which of the two activated CD8 T cell subsets contains the IFN-γ-producing cells. Figure 5A shows a flow cytometry example of the IFN-γ production of the gated CD8 T cell population after dengue virus peptide stimulation, and Fig. 5B shows the frequency of IFN-γ-producing CD8 T cells in individual patients after stimulation with each of the dengue virus protein peptide pools. Consistent with other studies (6, 29), the breadth of the response was diverse between individual patients, but for the most part, NS3-specific responses were most dominant (Fig. 5B, pie chart). Interestingly, the cumulative frequency of the total CD8 T cells producing IFN-γ in response to a combination of all the peptide pools derived from each of the 10 dengue virus proteins was rather low (range, 0.28% to 2.13% of the CD8 T cells). This low frequency of IFN-γ-producing cells was rather surprising considering the massive expansion and activation of the CD8 T cell subsets that was observed in the dengue patients. We found that the small numbers of IFN-γ-producing CD8 T cells preferentially segregate into the HLA-DR^+^ CD38^+^ double-positive CD8 T cell subset (Fig. 5C and D). From these results, we conclude that, although the IFN-γ cytokine-producing cells were present in both activated cell populations, they were preferentially enriched in the HLA-DR^+^ CD38^+^ double-positive population. However, the total IFN-γ-producing cells accounted for only a minute fraction of these massively expanding cytotoxic effector CD8 T cells.

### Transcriptomic analysis of CD8 T cells during dengue virus infection.

**FIG 6:**
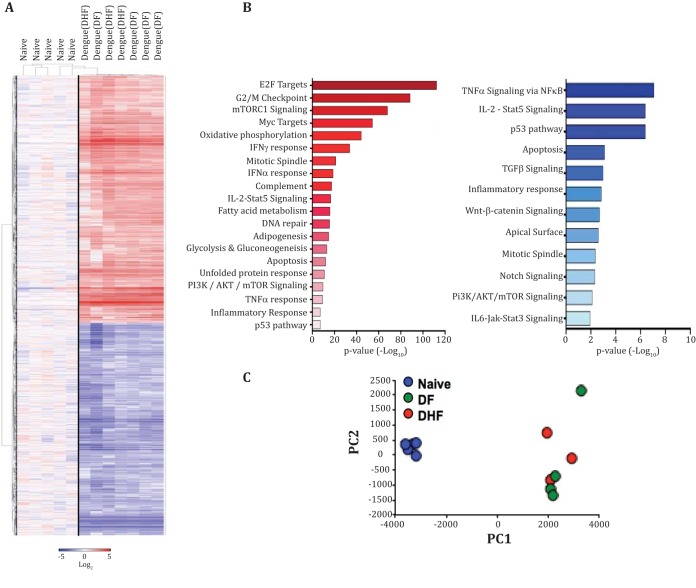

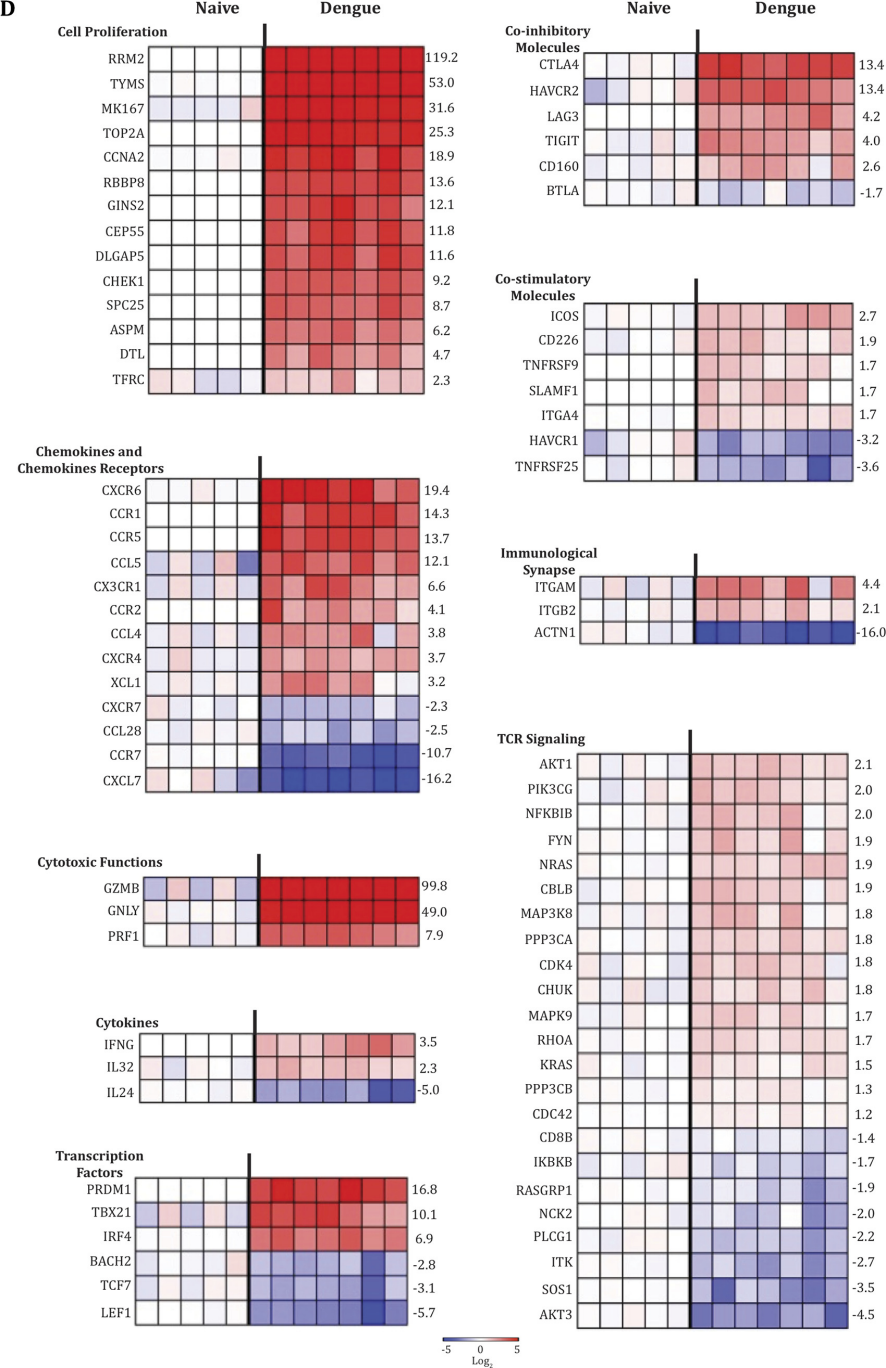
Transcriptomic analysis of CD8 T cells during dengue virus infection. (A) HCL showing 4,006 genes that were significantly upregulated (red) or downregulated (blue) in HLA-DR^+^ CD38^+^ CD8 T cells during dengue virus infection from seven patients (dengue, DF, and DHF) compared with CCR7^+^ CD45RA^+^ CD8 T cells from five healthy donors (Naive) (*P* ≤ 0.01; *t* test). Gene expression values were transformed into log_2_ scale. (B) Potential biological pathways containing genes that were at least 2-fold up- or downregulated in HLA-DR^+^ CD38^+^ CD8 T cells during dengue virus infection. The pathways were defined by the Hallmark Gene Sets from the Broad Institute's MSigDB. (C) Scatter plot showing PCA of identical gene sets used in the HCL analysis in panel A. The PCA was from the 4,006 genes that were significantly expressed compared to the naive cells (*P* ≤ 0.01; *t* test). (D) Heat maps showing selected genes that were significantly different from naive cells (*P* ≤ 0.01; *t* test). The genes are indicated on the left, and fold changes of the average expression value of the dengue group from that of the naive group are shown on the right.

Although at least three studies have examined gene expression profiles of blood or PBMCs in dengue patients (22–24), so far, no study has reported gene expression profiles of sorted CD8 T cells from dengue patients. To gain further insight into the characteristics of these activated CD8 T cells, we performed microarray analysis. HLA-DR^+^ CD38^+^ CD8 T cells were sorted from the PBMCs of seven dengue patients from Siriraj Hospital in Bangkok, Thailand. These patients were diagnosed with either mild dengue (DF) or severe dengue (DHF). By comparison with the sorted naive (CCR7^+^ CD45RA^+^) CD8 T cells, we found that these activated CD8 T cells from dengue patients showed global transcriptional changes across multiple pathways (Fig. 6A and B), the details of which are provided in theTable 2. PCA showed that there was no striking difference between the gene expression profiles of the activated CD8 T cells from DF versus those from DHF patients (Fig. 6C). Figure 6D shows selected genes of interest. The strong upregulation of genes involved in cell proliferation (MKI67, TOP2A, and CHEK1 genes) strengthened our phenotypic and quantitative analyses presented above and reinforces the idea that these cells were not simply a result of bystander activation but had been actively undergoing massive levels of antigen-driven proliferation *in vivo*. Consistent with the strong tissue-homing (CXCR6, CCR1, CCR5, CX3CR1, and XCL1) and cytotoxic-effector phenotypes described above, the expression of several genes involved in tissue homing were strongly altered, and the genes involved in cytotoxic-effector functions (GZMB, GNLY, and PRF1 genes) were massively upregulated. In contrast, the IFN-γ gene transcripts were moderately upregulated. Interestingly, these activated CD8 T cells from dengue patients showed signs of overwhelming antigenic stimulus *in vivo* that is often expected to lead to cytokine functional exhaustion via negative costimulation and TCR signaling attenuation. This is evidenced by upregulation of transcripts corresponding to a wide range of negative costimulatory molecules (PRDM1, CTLA4, HAVCR2, LAG3, TIGIT, and CD160). Additionally, we observed that these cells heavily downregulated the expression of ACTN1, a key component involved in actin polymerization and formation of the supramolecular activation cluster of the immunological synapse. These activated CD8 T cells also downregulated the expression of several other genes involved in T cell receptor signaling amplification (AKT3, SOS1, ITK, PLCG1, NCK2, and RASGRP1 genes).

**TABLE 2:** Comparison of sorted naive CD8 T cells and HLA-DR^+^ CD38^+^ CD8 T cells^*a*^

Gene set name	No. of genes in gene set (*K*)	Description	No. of genes in overlap (*k*)	*k*/*K*	*P* value	FDR *q* value
Upregulated genes^*b*^						
HALLMARK_E2F_TARGETS	200	Genes encoding cell cycle-related targets of E2F transcription factors	106	0.5300	4.27E−113	2.14E−111
HALLMARK_G2M_CHECKPOINT	200	Genes involved in the G2/M checkpoint, as in progression through the cell division cycle	91	0.4550	4.07E−89	1.02E−87
HALLMARK_MTORC1_SIGNALING	200	Genes upregulated through activation of mTORC1 complex	77	0.3850	1.36E−68	2.27E−67
HALLMARK_MYC_TARGETS_V1	200	Subgroup of genes regulated by MYC, version 1.	67	0.3350	4.55E−55	5.69E−54
HALLMARK_OXIDATIVE_PHOSPHORYLATION	200	Genes encoding proteins involved in oxidative phosphorylation	59	0.2950	5.68E−45	5.68E−44
HALLMARK_INTERFERON_GAMMA_RESPONSE	200	Genes upregulated in response to IFNG (GeneID, 3458)	50	0.2500	1.93E−34	1.61E−33
HALLMARK_MITOTIC_SPINDLE	200	Genes important for mitotic-spindle assembly	38	0.1900	6.43E−22	4.6E−21
HALLMARK_INTERFERON_ALPHA_RESPONSE	97	Genes upregulated in response to IFN-α proteins	26	0.2680	2.1E−19	1.31E−18
HALLMARK_ALLOGRAFT_REJECTION	200	Genes upregulated during transplant rejection	34	0.1700	3.52E−18	1.76E−17
HALLMARK_COMPLEMENT	200	Genes encoding components of the complement system, which is part of the innate immune system	34	0.1700	3.52E−18	1.76E−17
HALLMARK_ESTROGEN_RESPONSE_LATE	200	Genes defining late response to estrogen	33	0.1650	2.77E−17	1.15E−16
HALLMARK_IL2_STAT5_SIGNALING	200	Genes upregulated by STAT5 in response to IL-2 stimulation	33	0.1650	2.77E−17	1.15E−16
HALLMARK_FATTY_ACID_METABOLISM	158	Genes encoding proteins involved in metabolism of fatty acids	29	0.1835	1.22E−16	4.69E−16
HALLMARK_DNA_REPAIR	150	Genes involved in DNA repair	28	0.1867	2.58E−16	9.23E−16
HALLMARK_ADIPOGENESIS	200	Genes upregulated during adipocyte differentiation (adipogenesis)	31	0.1550	1.54E−15	5.14E−15
HALLMARK_GLYCOLYSIS	200	Genes encoding proteins involved in glycolysis and gluconeogenesis	29	0.1450	7.37E−14	2.3E−13
HALLMARK_ANDROGEN_RESPONSE	101	Genes defining response to androgens	21	0.2079	1.47E−13	4.32E−13
HALLMARK_CHOLESTEROL_HOMEOSTASIS	74	Genes involved in cholesterol homeostasis	18	0.2432	4.53E−13	1.26E−12
HALLMARK_UV_RESPONSE_UP	158	Genes upregulated in response to UV radiation	25	0.1582	5.14E−13	1.35E−12
HALLMARK_APOPTOSIS	161	Genes mediating programmed cell death (apoptosis) by activation of caspases	25	0.1553	7.95E−13	1.99E−12
HALLMARK_HYPOXIA	200	Genes upregulated in response to low oxygen levels (hypoxia)	27	0.1350	3E−12	7.14E−12
HALLMARK_UNFOLDED_PROTEIN_RESPONSE	113	Genes upregulated during unfolded protein response, a cellular stress response related to the endoplasmic reticulum	20	0.1770	1.29E−11	2.94E−11
HALLMARK_PI3K_AKT_MTOR_SIGNALING	105	Genes upregulated by activation of the PI3K/AKT/mTOR pathway	18	0.1714	2.35E−10	5.1E−10
HALLMARK_TNFA_SIGNALING_VIA_NFKB	200	Genes regulated by NF-κB in response to TNF (GeneID, 7124)	24	0.1200	5.64E−10	1.17E−9
HALLMARK_XENOBIOTIC_METABOLISM	200	Genes encoding proteins involved in processing of drugs and other xenobiotics	22	0.1100	1.47E−8	2.94E−8
HALLMARK_INFLAMMATORY_RESPONSE	200	Genes defining inflammatory response	21	0.1050	6.98E−8	1.29E−7
HALLMARK_P53_PATHWAY	200	Genes involved in p53 pathways and networks	21	0.1050	6.98E−8	1.29E−7
HALLMARK_SPERMATOGENESIS	135	Genes upregulated during production of male gametes (sperm), as in spermatogenesis	16	0.1185	4.83E−7	8.62E−7
HALLMARK_MYC_TARGETS_V2	58	Subgroup of genes regulated by MYC, version 2	10	0.1724	2.22E−6	3.83E−6
HALLMARK_REACTIVE_OXIGEN_SPECIES_PATHWAY	49	Genes upregulated by reactive oxygen species (ROS)	9	0.1837	4.14E−6	6.9E−6
HALLMARK_ESTROGEN_RESPONSE_EARLY	200	Genes defining early response to estrogen	18	0.0900	5.43E−6	8.48E−6
HALLMARK_HEME_METABOLISM	200	Genes involved in metabolism of heme (a cofactor consisting of iron and porphyrin) and erythroblast differentiation	18	0.0900	5.43E−6	8.48E−6
HALLMARK_PROTEIN_SECRETION	96	Genes involved in protein secretion pathway	12	0.1250	7.34E−6	1.11E−5
HALLMARK_PEROXISOME	104	Genes encoding components of peroxisome	11	0.1058	8.38E−5	1.23E−4
HALLMARK_UV_RESPONSE_DN	144	Genes downregulated in response to UV radiation	13	0.0903	1.03E−4	1.46E−4
HALLMARK_IL6_JAK_STAT3_SIGNALING	87	Genes upregulated by IL-6 (GeneID, 3569) via STAT3 (GeneID, 6774), e.g., during acute-phase response	8	0.0920	1.91E−3	2.65E−3
HALLMARK_BILE_ACID_METABOLISM	112	Genes involved in metabolism of bile acids and salts	9	0.0804	2.6E−3	3.51E−3
HALLMARK_APICAL_JUNCTION	200	Genes encoding components of apical-junction complex	11	0.0550	1.58E−2	2.02E−2
HALLMARK_EPITHELIAL_MESENCHYMAL_TRANSITION	200	Genes defining epithelial-mesenchymal transition, as in wound healing, fibrosis, and metastasis	11	0.0550	1.58E−2	2.02E−2
HALLMARK_APICAL_SURFACE	44	Genes encoding proteins overrepresented on the apical surfaces of epithelial cells, e.g., important for cell polarity (apical area)	4	0.0909	2.68E−2	3.35E−2
Downregulated gen**es**^*c*^						
HALLMARK_TNFA_SIGNALING_VIA_NFKB	200	Genes regulated by NF-κB in response to TNF (GeneID, 7124)	19	0.0950	8.59E−8	4.3E−6
HALLMARK_IL2_STAT5_SIGNALING	200	Genes upregulated by STAT5 in response to IL-2 stimulation	18	0.0900	4.18E−7	6.97E−6
HALLMARK_P53_PATHWAY	200	Genes involved in p53 pathways and networks	18	0.0900	4.18E−7	6.97E−6
HALLMARK_ESTROGEN_RESPONSE_EARLY	200	Genes defining early response to estrogen	17	0.0850	1.92E−6	2.4E−5
HALLMARK_UV_RESPONSE_DN	144	Genes downregulated in response to UV radiation	13	0.0903	1.6E−5	1.6E−4
HALLMARK_HYPOXIA	200	Genes upregulated in response to low oxygen levels (hypoxia)	13	0.0650	4.46E−4	3.71E−3
HALLMARK_APOPTOSIS	161	Genes mediating programmed cell death (apoptosis) by activation of caspases	11	0.0683	7.95E−4	5.68E−3
HALLMARK_TGF_BETA_SIGNALING	54	Genes upregulated in response to TGFB1 (GeneID, 7040)	6	0.1111	1.06E−3	6.66E−3
HALLMARK_INFLAMMATORY_RESPONSE	200	Genes defining inflammatory response	12	0.0600	1.45E−3	7.92E−3
HALLMARK_ANDROGEN_RESPONSE	101	Genes defining response to androgens	8	0.0792	1.58E−3	7.92E−3
HALLMARK_WNT_BETA_CATENIN_SIGNALING	42	Genes upregulated by activation of WNT signaling through accumulation of beta catenin CTNNB1 (GeneID, 1499)	5	0.1190	2.04E−3	9.26E−3
HALLMARK_APICAL_SURFACE	44	Genes encoding proteins overrepresented on the apical surfaces of epithelial cells, e.g., important for cell polarity (apical area)	5	0.1136	2.51E−3	1.05E−2
HALLMARK_MITOTIC_SPINDLE	200	Genes important for mitotic-spindle assembly	11	0.0550	4.38E−3	1.68E−2
HALLMARK_NOTCH_SIGNALING	32	Genes upregulated by activation of Notch signaling	4	0.1250	4.8E−3	1.71E−2
HALLMARK_PI3K_AKT_MTOR_SIGNALING	105	Genes upregulated by activation of the PI3K/AKT/mTOR pathway	7	0.0667	7.84E−3	2.61E−2
HALLMARK_IL6_JAK_STAT3_SIGNALING	87	Genes upregulated by IL-6 (GeneID, 3569) via STAT3 (GeneID, 6774), e.g., during acute-phase response	6	0.0690	1.15E−2	3.55E−2
HALLMARK_APICAL_JUNCTION	200	Genes encoding components of apical-junction complex	10	0.0500	1.21E−2	3.55E−2

a CD8 T cells from dengue patients show global transcriptional changes across multiple pathways. FDR, false discovery rate.

b Collection, H; no. of overlaps shown, 40; no. of gene sets in collection, 50; no. of genes in comparison (*n*), 1,191; no. of genes in universe (*N*), 45,956.

c Collection, H; no. of overlaps shown, 17; no. of gene sets in collection, 50; no. of genes in comparison (*n*), 992; no. of genes in universe (*N*), 45,956.

**FIG 7:**
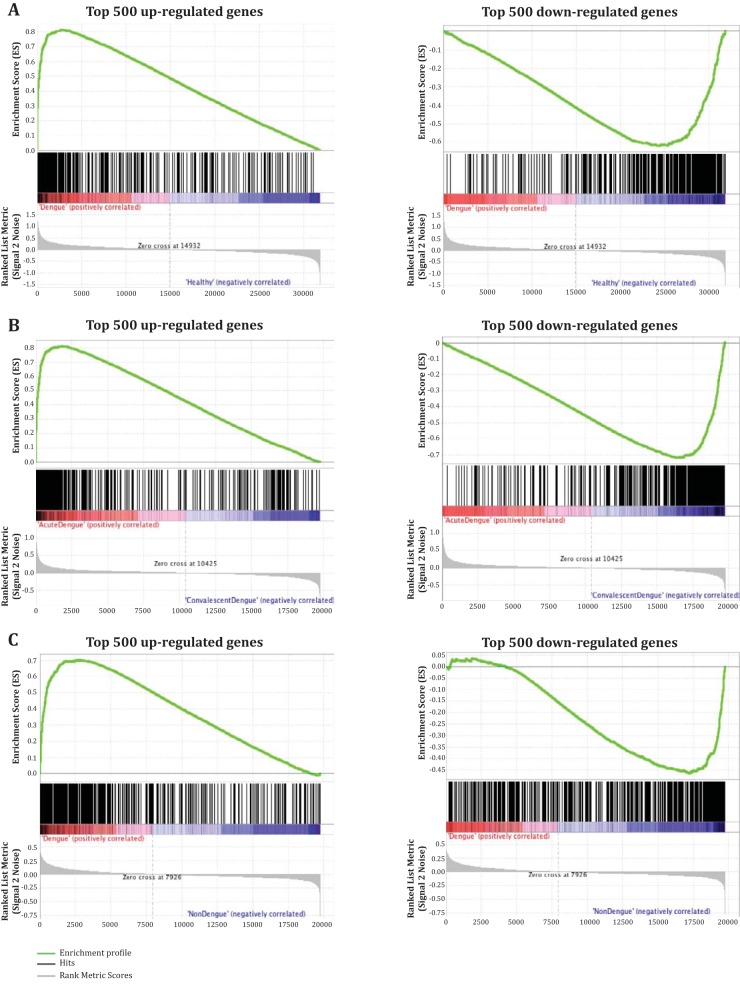

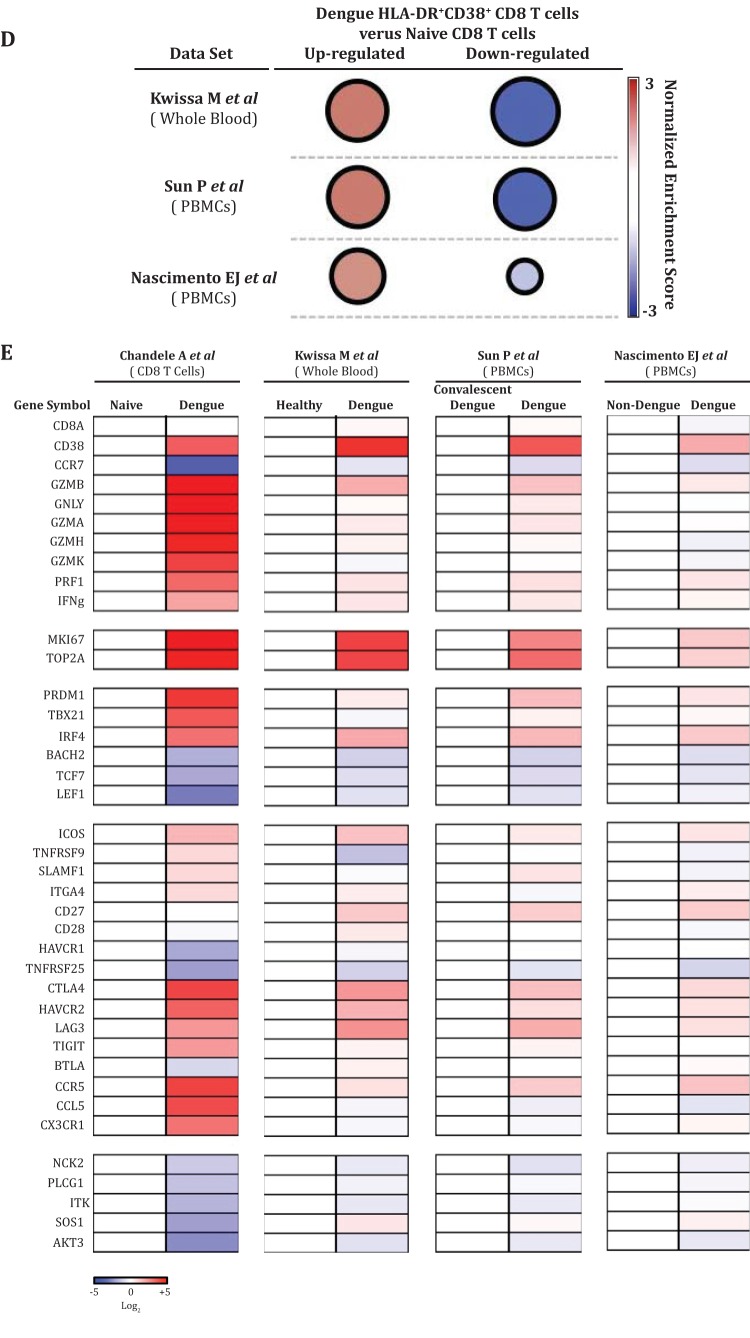
Comparative analysis of global gene expression profiles of CD8 T cells. (A to C) GSEA of the 500 most up- and downregulated genes from HLA-DR^+^ CD38^+^ CD8 T cells compared with GSE51808 (A) (22), GSE43777 (B) (23), and GSE18090 (C) (24) data sets. (D) Diagram summarizing the GSEA comparison of the top 500 up- and downregulated genes in HLA-DR^+^ CD38^+^ CD8 T cells during the acute phase of dengue infection with other published data sets. The sizes of the circles represent the proportions of overlapping genes in the leading-edge subset. (E) Heat map showing the expression of selected genes commonly expressed in CD8 T cells (A. Chandele et al.) in other data sets from M. Kwissa et al. (22), P. Sun et al. (23), and E. J. Nascimento et al. (24).

We also compared the gene expression profile of the sorted CD8 T cells from our study with previously published gene expression data sets of total PBMCs or whole blood from dengue patients from Thailand, Venezuela, and Brazil (22–24). The GSEA showed that the 500 most upregulated genes identified from our sorted HLA-DR^+^ CD38^+^ CD8 cells positively correlated with upregulated genes during dengue virus infection compared to healthy controls or non-dengue infection from other studies. The normalized enrichment scores (NES) of this comparison ranged from 1.5 to 1.7, and about 60% of the genes in the leading-edge subset overlapped (Fig. 7A to C). Similar to the upregulated genes, many of the 500 most downregulated genes identified from our sorted CD8 T cells were also downregulated compared with other data sets. NES between −1.2 and −1.8 with 32 to 62% overlapping in the leading edge between the subsets were identified. Moreover, we found similar trends of gene expression in many of the genes that belong to the CD8 T cell signature in the other three studies of PBMCs or whole blood from dengue patients (Fig. 7D to E). These results together show that this massive expansion and activation of CD8 T cells in these dengue patients forms an overwhelming proportion of the total dengue immune response and is strikingly similar in dengue patients from different geographical regions. Moreover, our gene expression results suggest these highly activated, massively proliferating, antigen-specific, cytotoxic CD8 T cells in the dengue patients may have received overwhelming antigen stimulus *in vivo* and, as a result, altered several molecular pathways that may contribute to suboptimal stimulation through TCR signaling insufficiencies.

### IFN-γ dysfunction of CD8 T cell subsets during acute dengue disease.

To examine whether the lack of IFN-γ production from a vast majority of these activated CD8 T cell subsets is indeed related to signaling insufficiencies suggested by our transcriptomics analysis or related to intrinsic defects in IFN-γ gene transcription and translation, we compared the IFN-γ production levels in the CD8 T cell subsets by stimulating them with TCR-dependent (anti-CD3^+^ CD28) and independent (PMA plus ionomycin) polyclonal stimuli.

**FIG 8:**
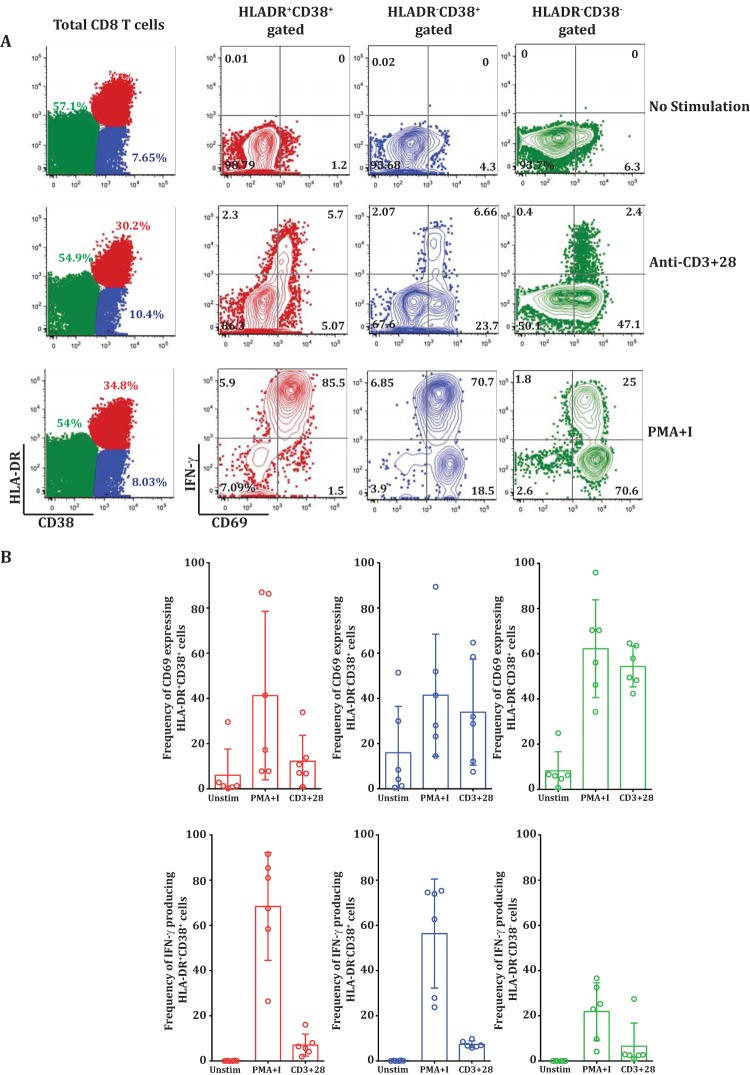
TCR stimulation-dependent IFN-γ dysfunction of CD8 T cells during acute dengue disease. (A) PBMCs from dengue fever patients were cultured *ex vivo* without stimulation (top row) or with anti-CD3/28 (middle row) or PMA plus ionomycin (bottom row). The plots on the left show surface staining of HLA-DR (*y* axis) and CD38 (*x* axis) on total gated CD8 T cells. The right three columns show individual CD8 T cell subsets, HLA-DR^+^ CD38^+^ (red), HLA-DR^−^ CD38^+^ (blue), and HLA-DR^−^ CD38^−^ (green), analyzed for CD69 (*x* axis) and IFN-γ (*y* axis). (B) Scatter plots comparing CD69 expression (top) and IFN-γ production (bottom) in HLA-DR^+^ CD38^+^ (red), HLA-DR^−^ CD38^+^ (blue), and HLA-DR^−^ CD38^−^ (green) CD8 T cell subsets treated with PMA plus ionomycin or anti-CD3 plus anti-CD28.

By polyclonal TCR-dependent stimulus (Fig. 8A, middle row, 4th plot), the vast majority of HLA-DR^−^ CD38^−^ double-negative cells (which are expected to be enriched in mostly naive cells) robustly upregulated CD69, and as expected, very few of them made IFN-γ. In contrast, the activated CD8 T cell subsets from the same patient were inefficient in both CD69 upregulation and IFN-γ production (Fig. 8A, middle row, 2nd and 3rd plots). Similar trends were observed for multiple patients (Fig. 8B). This result suggested that the vast majority of these massively expanding HLA-DR^+^ CD38^+^ double-positive and HLA-DR^−^ CD38^+^ single-positive subsets neither upregulated CD69 nor made IFN-γ when stimulated with a polyclonal TCR-dependent stimulus *in vitro*. However, when stimulated with a strong TCR-independent stimulus (PMA plus ionomycin), almost all the members of the activated HLA-DR^+^ CD38^+^ subset upregulated CD69 and made IFN-γ.

From these results, we conclude that these massively expanding, highly activated, cytotoxic effector CD8 T cells in dengue fever patients are indeed capable of making IFN-γ upon stimulation *in vitro* with a strong TCR-independent stimulus but do not make it with TCR-dependent stimuli, probably due to TCR signaling insufficiencies, as suggested by our transcriptomic analysis.

## DISCUSSION

Our study provides a comprehensive description of the two major subsets (HLA-DR^+^ CD38^+^ and HLA-DR^−^ CD38^+^) of CD8 T cells during dengue fever disease. We show that both subsets expand massively, but the double-positive HLA-DR^+^ CD38^+^ subset is much more robust in overall expansion and is equipped with a full spectrum of characteristics indicative of strong antigen-driven proliferation, tissue surveillance, and cytotoxic-effector functions.

These activated CD8 T cell subsets comprise a huge fraction of PBMCs during the acute febrile phase of dengue disease. From our results, we infer that on average, a dengue-infected child 10 years of age is expected to harbor about 10^6^ to 10^9^ of these highly activated CD8 T cells in the blood circulation alone, without accounting for the cells that are likely to have trafficked to inflammatory tissues. This represents a huge expansion of CD8 T cells during the febrile phase of dengue disease. The expansion of the CD8 T cells that we have seen in these dengue patients appears to be strikingly higher than the expansion reported in other human flavivirus infections, such as yellow fever and tick-borne encephalitis, or respiratory infections, such as influenza or respiratory syncytial virus infection (30–33). Moreover, the level of CD8 T cell expansion that we have seen in these dengue patients is somewhat similar to the expansion in human infections with other hemorrhagic-fever viruses, such Ebola virus and Puumala virus (34, 35). Although our study could not address whether this expansion would differ with the infecting dengue serotype because a majority of the patients recruited for the study in Delhi and successfully serotyped were DENV-2, the dominance of DENV-3 or DENV-4 in our Thailand patients suggest that the expansion is likely to be robust in patients infected with different dengue virus serotypes.

Considering that these dengue fever patients still harbor viral antigen at the time of clinical presentation, these massively expanding CD8 T cells are expected to cause a cytokine storm if they retain their ability to make IFN-γ *in vivo*. Interestingly, we found that very few of these massively expanding CD8 T cells derived from the dengue patients with febrile illness were capable of making IFN-γ *in vitro*, even when stimulated with dengue peptide pools spanning the entire proteome.

This *in vitro* IFN-γ unresponsiveness of the vast majority of these highly activated effector CD8 T cells is somewhat reminiscent of the T cell exhaustion seen under conditions of prolonged antigenic stimulus in chronic viral infections and closely resembles the “stunned” phenotype reported in the febrile phase of other acute infections, such as HIV infection and viral hepatitis (36, 37). In chronic infections, the exhausted CD8 T cells first lose IL-2 production, followed by tumor necrosis factor alpha (TNF-α) and finally IFN-γ production (38, 39). Our study was not designed to comprehensively test an extensive array of these other cytokines. However, it is worth pointing out that (i) in a limited number of experiments, we failed to detect any IL-2 production from these activated CD8 T cells after dengue virus peptide stimulation *in vitro* and (ii) from the other studies, we infer that the frequency of TNF-α-producing cells is also likely to be very low in dengue patients, on the order of 0.1 to 3% of the total CD8 T cells, which is not substantially different from the low frequency of IFN-γ-producing cells that we have seen in our dengue patient cohort (5).

An understanding of the mechanism by which these cells lose their capacity to produce cytokine is important and will potentially open novel therapeutic avenues for dampening inflammation, especially in infections like dengue. The IFN-γ cytokine repression in exhausted CD8 T cells in chronic infections is achieved by gene silencing at the transcriptional/translational levels through epigenetic gene methylation programs that are inherited between successive cell divisions. These programs are further regulated by the PD-1-inhibitory pathway and various regulatory proteins that induce and maintain cytokine exhaustion (40, 41). Because our phenotypic and transcriptomic analyses showed that these activated CD8 T cell subsets from dengue patients upregulate several negative costimulatory molecules, such as PD-1, Lag3, KLRG1, CTLA-4, and CD160, one would suspect that these cells may be subjected to IFN-γ repression in a manner similar to that of the exhausted CD8 T cells in chronic infections. Interestingly, our transcriptomic analysis indicated that these cells also downregulated several other key pathways involved in TCR signaling, amplification, and synapse. Prominent among these are AKT3, which is critical for costimulation via CD28/ICOS; SOS, RASGRP1, NCK2 and ITK, which are involved in early TCR signaling amplification through the adapter protein SLP-76; and ACTN1, which is a key molecule involved in the formation of the supramolecular activation cluster of the immunological synapse and sustained TCR signaling via actin polymerization. Based on these results, we wondered whether the lack of IFN-γ production in these cells was truly due to IFN-γ gene silencing, as observed in chronic infections, or to TCR signaling insufficiencies, as revealed by our transcriptomic analysis. To test these possibilities, we compared IFN-γ production after polyclonal TCR-independent (PMA plus ionomycin) or TCR-dependent (anti-CD3/28) stimulus. Our observation that these activated cells failed to upregulate CD69 or produce IFN-γ when stimulated by a polyclonal TCR-dependent stimulus but robustly upregulated CD69 and also produced IFN-γ when stimulated by a strong TCR-independent stimulus suggested that the cells are indeed capable of making IFN-γ but do not make it due the TCR signaling insufficiencies. Thus, the mechanism of IFN-γ unresponsiveness by these CD8 T cells in dengue fever illness is likely to be different from the mechanism that contributes to IFN-γ exhaustion in chronic infections such as HIV, where in T cells IFN-γ is reduced due to IFN-γ gene repression in CD8 T cells (38).

However, from these data, we cannot conclude whether a majority of the CD8 T cells were incapable of producing IFN-γ *in vivo*. Indeed, most dengue cases from studies performed in other parts of the world (42) and our Indian cohort had some IFN-γ in the plasma (28). This suggests that, perhaps, these cells and/or other immune cells were making IFN-γ *in vivo* prior to the arrival of the patient at the clinic, but a vast majority of them may have lost the *in vitro* IFN-γ production capacity by the time the patient experienced clinical symptoms and presented to the clinic. This hypothesis is further strengthened by our transcriptomic analysis showing that the activated CD8 T cells derived from these dengue patients upregulated IFN-γ-induced genes (Fig. 6B). Therefore, we predict that these activated CD8 T cells would have had the ability to produce IFN-γ *in vivo* but a vast majority of them may have acquired signaling insufficiencies in parallel with clonal expansion by the time the patient developed clinical febrile illness.

Taken together, our studies for the first time provide a comprehensive description of the phenotypes, functions, and molecular profiles of two major subsets of CD8 T cells that expand massively during the febrile phase of dengue disease. Both subsets acquire strong cytotoxic effector phenotypes and tissue-homing characteristics, with HLA-DR^+^ CD38^+^ being more robust in these qualities. Despite this strong cytotoxic-effector phenotype, the vast majority of these cells do not produce IFN-γ when stimulated with dengue peptides *in vitro*. Our studies reveal that this IFN-γ unresponsiveness is not due to intrinsic defects in IFN-γ gene transcription/translation but is related to TCR signaling insufficiencies. The IFN-γ unresponsiveness acquired during the massive antigen-driven clonal expansion is likely to ensure that these cells do not cause excessive inflammation at the time that their numbers are high during the febrile phase of dengue disease. These results have implications for understanding the mechanisms determining the balance between CD8 T cell-mediated protection and pathology during the febrile phase of dengue disease.
